# Clinical Evidence on a Novel Macrohybrid Design Dental Implant with 12° Angled Platform: A Systematic Review

**DOI:** 10.3390/ma15145011

**Published:** 2022-07-19

**Authors:** Andrea Galve-Huertas, Maria José Zilleruelo-Pozo, Susana García-González, Octavi Ortíz-Puigpelat, Federico Hernández-Alfaro, Samir Aboul-Hosn Centenero

**Affiliations:** 1 Department of Oral and Maxillofacial Surgery, Universitat Internacional de Catalunya, 08017 Barcelona, Spain; mjosezilleruelo@gmail.com (M.J.Z.-P.); susanagarcia@uic.es (S.G.-G.); octaviortiz@uic.es (O.O.-P.); h.alfaro@uic.es (F.H.-A.); samir@uic.es (S.A.-H.C.); 2 Staff Member of the Oral and Maxillofacial Surgery Department, Hospital Clinic de Barcelona, 08036 Barcelona, Spain

**Keywords:** co-axis implant, implant angulation, success rate, marginal bone loss

## Abstract

**Background**: Immediate implant placement with immediate esthetics has become a more common procedure over time, though ensuring good emergence of the axis of the implant has been a challenge. A novel macroimplant design with an angled platform (Co-Axis^®^) has been developed to ensure exit of the head of the implant in the correct prosthetic position. A systematic literature review was carried to determine the survival rate and marginal bone loss associated with these implants. **Material and Methods**: An electronic and manual literature search was made in accordance with the PRISMA statement. The search strategy was limited to human studies, retrospective and prospective clinical trials, cross-sectional studies, and cohort studies reporting outcomes of a novel macrohybrid implant with a 12° angled implant connection. **Results**: Three articles met the inclusion criteria and were reviewed in the analysis. The estimated success rate was 95.9%. The global marginal bone loss was estimated to be −0.17 ± 0.58 mm in an environment characterized by great heterogeneity (I^2^ = 99%). The estimated mean implant stability was 69.6 ± 0.92 (ISQ). As only two studies provided the required information, it was not possible to determine publication bias. Lastly, mean recession was estimated to be practically zero (0.06 ± 0.23 mm), with great heterogeneity. **Conclusions**: Within the limitations of this systematic review, it can be affirmed that immediate implant treatment with Co-Axis^®^ implants shows a survival rate of 95.9% at one year of follow-up, with low marginal bone loss values, near-zero soft tissue recession, and favorable papilla index values. Nevertheless, the great heterogeneity of the data requires the findings to be interpreted with caution.

## 1. Introduction

Immediate implant placement with immediate esthetics has become a more common procedure since the early 2000s [1]. This strategy seeks to reduce treatment time and preserve soft tissue architecture, thereby ensuring better outcomes [2].

Three-dimensional (3D) implant positioning is currently mandatory in implant dentistry, as well as leaving a minimum 2 mm buccal gap from the implant and a 3–4 mm apical distance from the desired soft tissue margin. Nowadays, immediate implants are placed palatal as a way to avoid the proximity of the buccal wall and possible recessions [3].

Nonetheless, ensuring good emergence of the axis of the implant following these guidelines—especially in the anterior maxilla and in post-extraction sockets—has been a challenge [4]. There are a number of possible solutions to this problem: (1) Cemented restorations in the anterior zone can be used, but this may result in subgingival migration of cement and difficulties for removal. (2) Another option is the use of dynamic screws or the alternative of using a transepithelial abutment that can be angulated. Abutments of this kind pose some problems: high laboratory costs as well as more complex prosthetic rehabilitation; a weaker connection with more crown loosening and more screw factures; reduction in the prosthetic space [5]. Furthermore, in many cases, the abutment produces discoloration of the gums. When the gingiva is less than 2 mm thick, titanium abutments should be avoided in anterior areas. In effect, the thickness of the soft tissue seems to be the key factor underlying the influence of the abutment on the tone of the soft tissues [6]. (3) Lastly, an implant with an angled platform may be used [7].

A novel macroimplant design was introduced in 2007, involving an implant with a 12° angled platform [8]. The implant is positioned in the middle of the alveolus, which optimizes the available bone of the socket, while, at the same time, the angulated platform affords a perfect emergence profile in the correct position. In addition, the elimination of an angled abutment reduces the need for customized abutments, achieving the best emergence profile. Initially, the angulations were limited to 12° and 24° degrees, with only an external hexagon connection, though nowadays, internal conical connections are available, with angulations ranging to 12° [9].

Over time, many surgical devices involving new surgical techniques have been introduced. All these innovations need to be evaluated in order to establish their applicability and clinical evidence [10].

In this regard, as no systematic reviews on this novel implant have been found, we decided to carry out a systematic literature review to determine the survival rate and marginal bone loss associated with the use of these implants, and to assess the feasibility of this treatment alternative.

## 2. Materials and Methods

### 2.1. Protocol and Registration

This systematic review followed the Preferred Reporting Items for Systematic Review and Meta-Analysis (PRISMA) statement [11], and is registered at PROSPERO (Ref. CRD42020219028).

### 2.2. Search Strategy

**Figure 1:**
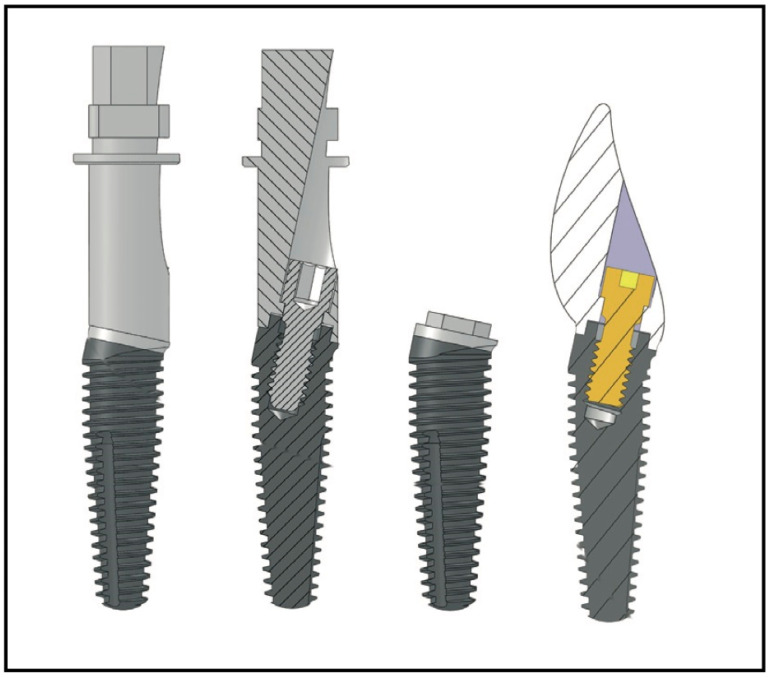
Co-Axis^®^ 12° implant.

The central question of the study was developed based on the PICOS [12] template: Does the novel macrohybrid implant with a 12° angled prosthodontic platform offer a good survival rate and acceptable marginal bone loss? (Figure 1).

Electronic and manual literature searches were made by two independent reviewers (MZ and AG) in the National Library of Medicine (Medline via PubMed) for articles published up until March 2021.

The following search terms were used: ((((((((((((“tapered implant”[All Fields]) OR (“conical implant”[All Fields])) OR (“conventional implant”[All Fields])) OR (“angled implant”[All Fields])) OR (“angulated implants”[All Fields])) OR (“co axis”[All Fields])) OR (“dental angulation”[All Fields])) OR (“implant angulation”[All Fields])) OR (“southern implants”[All Fields])) AND (“immediate implant”[All Fields])) OR (“postextraction implant”[All Fields])) OR (“immediate extraction sockets”[All Fields])) OR (“immediate loading”[All Fields]).

### 2.3. Eligibility Criteria

Articles were included in the systematic review if they met the following inclusion criteria:

1. Publications in English.
2. Human studies—retrospective and prospective clinical studies, cross-sectional studies, and cohort studies.
3. Reporting on outcomes of a novel implant with a 12° angled prosthodontic platform.
4. Articles including data on the survival rate of the implants and/or marginal bone loss at final follow-up.

The following exclusion criteria were established:

5. Studies reporting outcomes of a novel implant with 24° angled prosthodontic platform.
6. In vitro studies.
7. Animal studies.
8. Case series.

### 2.4. Study Selection

In the first phase of the systematic review, two reviewers (MZ and AG) analyzed all the identified titles and abstracts to assess eligibility for inclusion, based on the inclusion and exclusion criteria.

In a second stage, the full-text articles of all the studies selected in the first phase were retrieved and evaluated by both reviewers (MZ and AG) on an independent basis. Any disagreements between the investigators were resolved by discussion with two additional reviewers (SG and OO).

### 2.5. Data Extraction

The data analyses were performed independently by two reviewers (MZ and AG).

The following outcomes were extracted from each individual study: (1) author(s); (2) year of publication; (3) study design; (4) total number of patients; (5) total number of implants; (6) tooth replacement; (7) implant system; (8) length and diameter of the implant; (9) immediate implant procedure; (10) use of biomaterials; (11) flap or flapless design; (12) use of connective tissue grafting; (13) immediate loading procedure; (14) type of prosthesis; (15) duration of follow-up; (16) study outcomes (implant success, marginal bone loss, implant stability, clinical parameters, peri-implant tissue stability, esthetic outcomes, patient outcomes, and complications).

### 2.6. Data Analysis

The primary outcomes of the systematic review were: (a) implant success rate (SR) determined at baseline and at final follow-up (Albrektsson and Isidor 1994); (b) marginal bone level (MBL) changes measured using standardized intraoral radiographs (mesial and distal).

The secondary outcomes were: (a) implant stability measured by the Osstell implant stability quotient (ISQ) at implant placement and at final follow-up; (b) peri-implant tissue stability: papilla index according to Jemt 1997 and Hall 2007; (c) the most apical point of the mid-buccal gingival margin.

### 2.7. Risk of Bias

Two reviewers (MZ and AG) conducted the present study following the Strengthening the Reporting of Observational Studies in Epidemiology (STROBE) [13] statement and PRISMA [11] guidelines.

### 2.8. Quality of the Studies

For the nonrandomized studies, the Newcastle–Ottawa scale was used, allowing the evaluation of each publication included in the study, comparability of the cohorts, and the relationship between exposure and the studied outcome. Study quality scores were established, with a maximum score of 9 points.

### 2.9. Statistical Analysis

The raw success proportion was the first measure of effect to be estimated. The estimation of the meta-analysis was established on a random-effects model with a maximum likelihood estimator.

The meta-analysis to evaluate the total weighted average MBL was carried out using a random-effects model with a maximum likelihood estimator and a test based on the z-distribution and 95% confidence interval (95% CI).

A similar method was used for the rest of the secondary outcomes (stability, recession, and papilla).

The heterogeneity of the study was based on the I^2^ statistic (percentage of variability of the estimated effect that can be attributed to heterogeneity of the true effects) and the corresponding statistical test of nullity of Cochran’s Q. An I^2^ value >75% was interpreted as representing great heterogeneity. In articles reporting a 0% or 100% rate, the standard error was estimated using the Wilson method and the exact binomial formula to facilitate calculation of the heterogeneity indicators. Galbraith plots were used to analyze the contribution of the included studies to overall heterogeneity. In situations of significant heterogeneity, the source was explored using sensitivity analyses or subgroup analyses.

For selection bias, funnel plots were represented and the Egger test was performed.

The level of significance used in the analyses was 5% (α = 0.05). The meta-analysis was performed using the R 3.5.1 package (R Core Team (2018); R: A language and environment for statistical computing. R Foundation for Statistical Computing, Vienna, Austria; URL http://www.R-project.org/, accessed on 11 June 2021).

## 3. Results

### 3.1. Study Selection

**Figure 2:**
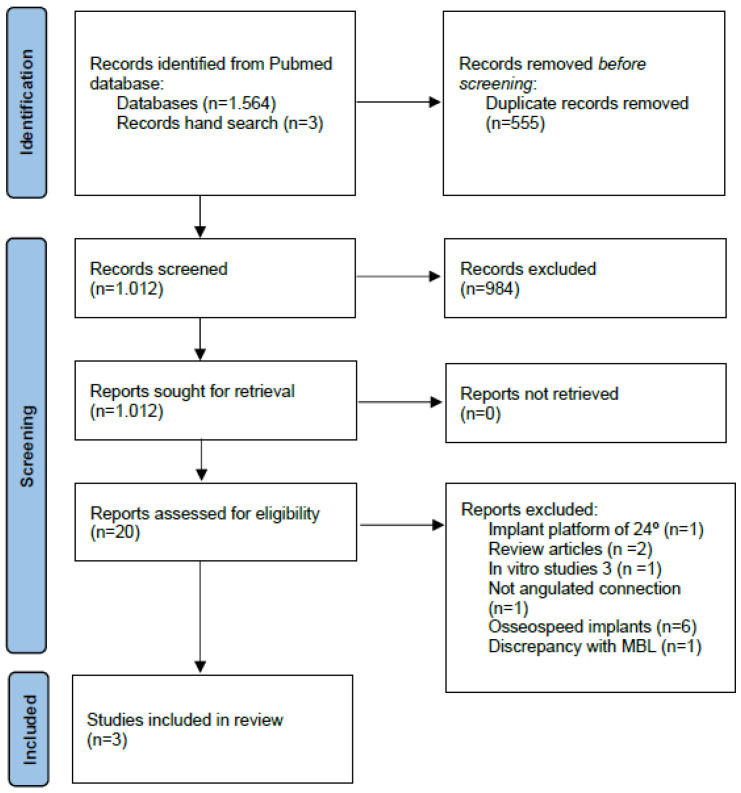
PRISMA flowchart of the screening process [17].

The initial screening yielded a total of 1564 articles. After evaluation of the abstract, 20 studies were subjected to full-text review. Of these twenty articles, three [14,15,16] met the inclusion criteria and were included in the analysis (Figure 2).

The article published by Van Weehaeghe et al. [18] was excluded, as it involved a 24° angled implant platform that was not considered comparable to the 12° angled platform. Case-series and pilot studies [19,20,21], review articles [22,23], and in vitro studies [8] were excluded, due to their low scientific evidence. In addition, one article was omitted because the data referring to bone loss were inadequate—measuring only buccal wall width and not mesial and distal bone loss [9]. In addition, 6 articles [24,25,26,27,28,29] were excluded because they investigated the OsseoSpeed^TM^ Profile implant with an inclined shoulder configuration, where the aim was not to correct emergence of the immediate implant but to match the vertical discrepancy of the alveolar ridge following the bone anatomy. Finally, one article did not use the angulated connection implant and was also excluded [1].

### 3.2. Characteristics of the Studies

#### 3.2.1. Intervention Category and Sample Characteristics

Three prospective clinical studies [14,15,16] met the inclusion criteria, involving a total of 57 patients and 60 implants, with a mean follow-up of 1–5 years.

The studies included implants in the pre-maxilla between 1.5 and 2.5. The implants were external Co-Axis 12° (Southern implant).

The study published by Brown et al. [14] in 2010 evaluated immediate placement and immediate restoration in fresh extraction sockets with a follow-up of one year. The article by Ma et al. 2018 [16] was the continuation of an earlier article with one-year data from Brown et al. [14]. The aim of the study was to report the 1- to 5-year clinical outcomes. Each immediate implant was placed without soft or hard tissue grafting, and in some cases, a flap was raised and provisionalization was delivered before the definitive crown.

On the other hand, Vandeweghe et al. 2011 [15] evaluated single implants in the pre-maxilla with immediate loading, no connective tissue graft, and no bone graft. The data of the included studies are summarized in Table 1.

#### 3.2.2. Outcomes Methodology

Assessment of the success rate was based on Albrektsson and Isidor, allowing 1.5 mm of bone loss during the first year [14,15], or categorizing success as a marginal bone loss of <1.8 mm [16].

Regarding the evaluation of MBL, the articles used standardized periapical radiographs with the implant-abutment junction in the mesial and distal part as the reference point, except for one article [12], which used the implant thread as reference in the mesial and distal part. The measurements of bone loss were calculated with a Peak Loupe scale under ×7 magnification [14,16] or using computer software.

The secondary outcome of implant stability was evaluated based on Osstell^TM^ (Integration Diagnostics, Göteborg, Sweden) at implant placement, baseline, and follow-up [14,16]. The additional secondary outcome of peri-implant tissue stability was assessed using the papilla index [30]. In turn, the apical gingival margin of the buccal mucosa of the implant crowns was evaluated from plaster models with digital calipers (accuracy 0.1 mm), with references to a line marked between the most apical middle buccal part and the gingival margins of implants adjacent to teeth [14,16] or using clinical photographs and an individual bite-fork for standardization. The pictures were calibrated and three lines were used through a chosen reference to measure the mesial and distal papilla and zenith, employing morphometric software [15].

### 3.3. Success Rate

**Figure 3:**
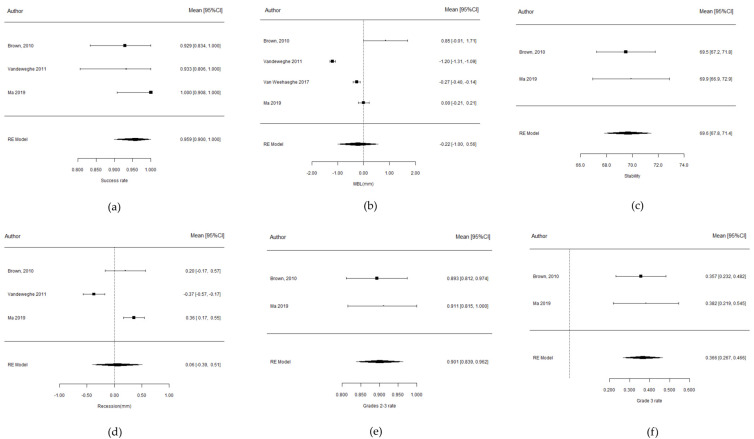
Forest plot showing the meta-analysis results referring to (**a**) success rate; (**b**) marginal bone level changes; (**c**) implant stability; (**d**) soft tissue recession; (**e**) papilla index grade 2–3; (**f**) papilla index grade 3 of Brown et al., 2010 [14], Ma et al., 2019 [16] and Vandeweghe et al., 2011 [18].

The three studies included provided information about the success rate [14,15,16], representing a global sample of 60 implants. These three studies reported similar rates, of the same order of magnitude (Figure 3a).

The estimated success rate was 95.9% (95% CI: 0.9–1.0), indicating that the treatment ensures a success rate of >90% with a probability of 97.5%. This estimated success rate was valid for one year of follow-up, as two of the studies [14,15] reported data at one year, while the study published by Brown [14] reported a 100% rate at 5 years—with the rate consequently also being assumed to be 100% at one year (Table 2).

The heterogeneity between studies represented 0% of the total variability (I^2^ = 0). The results of the Cochrane Q-test confirmed the results to be homogenous (*p* = 0.521) (Table 2).

**Figure 4:**
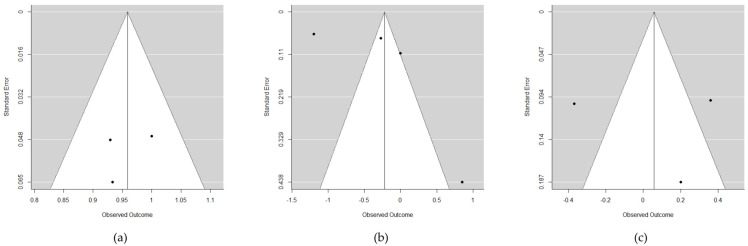
Funnel plots showing the risk of bias referring to (**a**) success rate; (**b**) marginal bone level changes; (**c**) soft tissue recession.

With only three studies analyzed, the Egger test was not very powerful, and although an absence of publication bias was reported (*p* = 0.585), the funnel plot (Figure 4a) evidenced very clear symmetry, as the bottom of the plot is the area corresponding to imprecise studies lacking any pattern, because all three articles had small sample sizes.

### 3.4. Marginal Bone Loss

Three studies provided information on marginal bone loss (MBL) [14,15,16] but referred to different periods of time. A recalculation was therefore made to obtain an annual marginal bone loss.

The mean global marginal bone loss was estimated to be −0.17 ± 0.58 mm (Figure 3b). The 95% confidence interval included 0, i.e., there was not enough statistical evidence (*p* = 0.767) to affirm that bone loss occurred. However, the estimates were obtained in an environment of great heterogeneity (I^2^ = 99%).

The funnel plot (Figure 4b) clearly reflected this great heterogeneity, as well as possible publication bias (*p* = 0.095). First, at the bottom right of the plot lies the study published by Brown [14], which was very imprecise due to its large standard deviation with an atypical bone gain. At the top of the plot, we find the other two studies, and one of them [15] appears isolated on the left, as it reports an atypical high marginal bone loss, which is not in line with the study published by Ma et al. [16].

### 3.5. Implant Stability

Only two studies afforded information on implant stability [14,16]. In both articles, the data corresponded to one year of follow-up, with very similar values.

The estimated mean implant stability was 69.6 ± 0.92 (95% CI: 67.8–71.4) (Figure 3c).

The homogeneity of both articles was in fact the total in terms of stability measured at one year (I^2^ = 0%; *p* = 0.836).

With only two articles, publication bias could not be adequately assessed using the Egger test.

### 3.6. Soft Tissue Recession

All three articles [14,16] reported results referring to soft tissue recession at one year. Two of the studies [14,15] reported gains, while the study of Ma et al. [16] reported soft tissue recession.

The mean recession was estimated to be practically zero (0.06 ± 0.23 mm), as a consequence of the trade-off between somewhat disparate individual results (Figure 3d).

As expected, the model warns of strong heterogeneity. The funnel plot (Figure 4c) showed separate articles around the vertical axis due to the heterogeneity of the estimated effects, though a certain symmetry was maintained (*p* = 0.785), and there were no signs of publication bias.

### 3.7. Papilla Index

This parameter was evaluated based on the criterion of Jemt et al. [30] The index comprises an ordinal grading of 5 levels measuring the amount of papilla present. Grades 2 and 3 correspond to the optimum condition (Figure 3e). Authors reported the percentage of evaluated sites at the mesial and distal level. Two sites per implant were thus evaluated, estimating the mean proportion of optimum sites measured. The estimated rate was 0.901 (90.1%) with a confidence interval of 0.5% (0.84–0.96). This would allow us to conclude that the treatment ensures a success rate of >84% with a probability of 97%. Table 3 evidences the existence of homogeneity between studies.

According to the results up to one year, the measurements progressed to grade 3 during the first year in the studies published by Brown et al. [14] and Ma et al. [16]. The estimated rate was 0.366 (36.6%) (95% CI: 0.27–0.47) (Figure 3f). This indicates that the studied implant treatment ensures a grade 3 papilla index at over 26.7% of the sites with a probability of 97.5%. Table 4 evidences the existence of homogeneity between the two studies.

## 4. Discussion

The present systematic review was carried out to analyze the survival and success rates reported in the literature in reference to a novel implant design, as well as the associated marginal bone level changes. As mentioned above, conduction of the present review was justified by the lack of systematic reviews on implants with an angled neck emergence. In effect, the main issue that emerges from the analysis of this systematic review is whether the Co-Axis^®^ implant affords acceptable bone stability, success, and survival. As it presents a unique body with an angled neck and a widening of the middle third, it is of interest to investigate such a novel implant.

Nowadays, the duration of implant placement is a crucial element, especially in the anterior zone where preservation of the soft tissue architecture is mandatory in order to secure good esthetic outcomes [31]. Immediate implant placement with immediate esthetics offers the possibility of maintaining soft tissue shape and of shortening the treatment time, thus defining this as a predictable treatment strategy [32]. Nonetheless, drilling for implant placement in a fresh extraction socket sometimes makes it difficult to follow the correct path of insertion, considering the ideal prosthetically guided implant position and available bone, with a view to securing primary stability. As a result, in many cases, the clinician must resort to other prosthetic solutions in order to resolve implant position issues.

In this systematic review, three articles met the final inclusion criteria and were analyzed. All three of them evaluated the use of Co-Axis^®^ implants comprising an external hexagon connection with an angulation of 12° in the anterior maxilla, with immediate provisionalization. No grafting of hard or soft tissues was performed in any of the studies. The estimated overall success rate for three of the four studies [14,16] was 95.9%. This is comparable to the results recorded in other studies, such as the systematic review by Galucci et al. [33], who obtained success rates ranging from 87 to 100% for immediate implants and with immediate provisionalization in straight implants. Immediate implants have a high survival rate. In the study carried out by Lee et al., out of 249 evaluated implants, five failed, yielding a 97.37% cumulative survival rate after 5 years of follow-up. This figure was slightly higher than that recorded in the present systematic review [34]. However, it should be noted that the articles included in our review used external connection implants, and this could influence the results obtained. Camps-Fonts et al. conduced a meta-analysis to determine whether the implant connection influences prosthodontic complications and implant survival. They found the internal connection to be associated to less bone loss and prosthodontic complications than the external connection, without compromising implant survival. Although it may seem that there is no influence upon the external connection and survival, the results must be interpreted with caution, as a number of uncontrolled factors could influence the results, such as implant diameter, design, and occlusal forces [35]. It therefore cannot be firmly concluded that there is a difference between these two connections. This is also evidenced in the article by Esposito et al., involving a 5-year randomized clinical trial in which 96 external connection implants were placed and only one failed, versus three failures out of 107 internal connection implants—the difference failing to reach statistical significance [36]. Similarly, in the literature, we identified a study in which immediate implants with external and internal connections were evaluated, recording survival rates of 97.7% with the external connections versus 97.5% with the internal connections. The difference was not statistically significant, thus likewise coinciding with the results of the present systematic review [37].

Finally, a favorable element is the fact that the three studies in this systematic review involved the same intervals and therefore presented great homogeneity, thus making the results highly comparable.

Regarding marginal bone loss, all the studies provided information on this parameter. The mean overall marginal bone loss was estimated to be −0.17 ± 0.48 mm, with no statistically significant differences. This constitutes acceptable bone loss for an observation period of less than a year. Concerning this variable, great heterogeneity was observed between studies, and the risk of bias was therefore higher. This could be associated with different follow-up periods within each study. In addition, all the studies used different time intervals to measure this outcome, and moreover adopted different methodological approaches—though all of them used standardized periapical radiographs and obtained measurements with different digital applications or even analogical analyses with a loupe and a scale [14]. These facts could explain the heterogeneity and higher risk of bias. In most of the articles included in the present study, immediate implants were placed without filling the gap, and bone loss was approximately 0.17 mm. However, in one study where immediate implants without gap filling were evaluated, the bone loss was found to be 0.28 mm [38]. Similar findings were obtained in the study by Park et al., who carried out a retrospective analysis of 242 implants and recorded an average bone loss of 0.28 mm. The observed bone loss values are in line with those recorded in previous studies [39]. In the same way as in the case of implant success, some comments must be made regarding bone loss. As has been mentioned, the articles included in the present study used external connection implants, and this type of connection is often associated with greater bone loss, as observed in the aforementioned article by Camps-Fonts et al., where bone loss was less pronounced in implants with an internal connection [35]. In this same line, the study carried out by Caricasulo et al. documented greater bone loss with external connections (1.32 mm) than with internal connections (1.20 mm). However, as mentioned above, some articles have reported no statistically significant differences between connections in terms of bone loss. It is therefore not possible to draw firm conclusions in this regard [36].

Finally, the peri-implant tissues were also evaluated with different measuring tools. In this regard, the studies of Ma et al. [16] and Brown et al. [14] used the papilla index method, while Vandeweghe et al. [15] used digital analysis of intraoral photographs. All these methodological differences make comparisons of the studies difficult.

Implant stability values were measured based on the Ostell^®^, as commented above. The mean overall stability value for this type of implant was estimated to be 69.6 ± 0.92. This outcome was reported by only two articles [14,16], though it indicates high stability, which is one of the key elements in assessing the success of immediate implants, and particularly the possibility of performing immediate esthetics [4].

As mentioned before, the preservation of soft tissue architecture is one of the objectives of immediate implant placement and provisionalization. Mean soft tissue recession was estimated to be almost zero in the present systematic review. Nonetheless, the results should be viewed with caution due to the high variability between studies. Such values may be attributed to the immediate esthetics and provisionalization performed in the two studies [14,15] that reported this outcome.

The papilla index was analyzed based on the Jemt index score at the mesial and distal level of the implant. This classification was described in 1997 [30], and is characterized by 5 levels: grade 0 = no papilla is present; grade 1 = less than half of the papilla is present; grade 2 = half or more of the height of the papilla is present; grade 3 = the papilla fills up the entire proximal space; grade 4 = the papillae are hyperplastic. Grades 2 and 3 are considered to reflect the optimum situation. In 90% of the cases, grade 2 or 3 was present, and the measurements were seen to improve to grade 3 within the first year. This indicates a favorable soft tissue outcome for this kind of implant over one year of follow-up.

The limitations of the present systematic review where the small number of studies that met the inclusion criteria referred to this type of angled neck implant, as well as the differences in study design, evaluation method, sample size, and duration of follow-up. Nonetheless, it was possible to perform a thorough analysis of important outcomes of these four studies. On the other hand, we identified no studies evaluating whether the design of this implant could cause problems in prosthetic rehabilitation. The idea of this type of implant is to facilitate placement at the center of the alveolus, and prosthetic emergence is corrected with the angled connection. However, if clinicians are not used to this type of implant, complications could develop during placement, resulting in incorrect prosthetic emergence. It therefore would be interesting to conduct a study on this issue.

## 5. Conclusions

Within the limitations of this systematic review, it can be concluded that immediate implant treatment with Co-Axis^®^ implants affords a 95.9% success rate after one year of follow-up, exhibiting no differences versus immediate implant treatment with conventional implants. In addition, low marginal bone loss values (−0.17) were found after one year of follow-up. Nonetheless, great heterogeneity was recorded, and the results therefore must be interpreted with caution. Regarding soft tissue recession, the recorded values were near zero, with favorable papilla index values. In conclusion, further studies on these types of angled neck implants are required, with improved methodological designs and larger sample sizes.

## Figures and Tables

**Table 1:** Characteristics of the studies included in the systematic review. Caption: *n*= number, CTG = connective tissue graft.

	Study Design	No. of Patients	No. of Implants	Tooth Replacement	Implant System	Length and Diameter (mm)	Immediate Implant	Biomaterial	Flap/Flapless	CTG	Immediate Provisionalization	Type of Prosthesis	Follow-Up (Months)	Outcomes
**Brown et al., 2010 [14]**	Prospective clinical study	27	28	15–25	Co-axis 12° Southern external connection	Length: 13–15 Diameter: 4–4.7	Yes	No	26 Flapless 2 Flap	No	Yes (without loading)	Screw-retained	12 (4 h provisional, 8 weeks definitive crown, 1 year follow-up)	Primary: (1) Implant success. (2) Marginal bone loss. Secondary: (1) Implant stability. (2) Peri-implant tissue stability. (3) Prosthodontic success.
**Vandeweghe et al., 2011 [18]**	Prospective clinical study	14	15	15–25	Co-axis 12° Southern external connection	Length: 10–13–15 Diameter: 4–5	No	No	Flap	No	Yes (with loading)	Screw-retained	12	(1) Marginal bone loss. (2) Implant survival and success. (3) Plaque and bleeding. (4) Soft tissue changes. (5) Patient satisfaction. (6) Esthetic outcomes. (7) Complications.
**Ma et al., 2019 [16]**	Prospective clinical study (single-arm)	16	17	15–25	Co-axis 12° Southern external connection	Not reported	Yes	No	Not reported	No	Yes (with loading)	Screw-retained	60 (4 h provisional, 8 weeks definitive, follow-up 5 years)	Primary: (1) Implant success. (2) Marginal bone loss. Secondary: (1) Implant stability. (2) Peri-implant tissue stability. (3) Prosthodontic success.

**Table 2:** Meta-analysis results referring to success rate. Caption: WMP = weighted mean prevalence; SE = standard error; CI = confidence interval; QH (*p*-value) = Cochrane Q of heterogeneity.

WMP	SE	95%CI	I^2^	Q_H_ (*p*-Value)	Egger (*p*-Value)
0.959	0.030	0.900–1.017	0.0%	0.521	0.585

**Table 3:** Meta-analysis results referring to papilla index grade 2–3. Caption: WMP = weighted mean prevalence; SE = standard error; CI = confidence interval; QH (*p*-value) = Cochrane Q of heterogeneity.

WMP	SE	95% CI	I^2^	Q_H_ (*p*-Value)	Egger (*p*-Value)
0.901	0.032	0.839–0.962	0.0%	0.778	-

**Table 4:** Meta-analysis results referring to papilla index grade 3. Caption: WMP = weighted mean prevalence; SE = standard error; CI = confidence interval; QH (*p*-value) = Cochrane Q of heterogeneity.

WMP	SE	95% CI	I^2^	Q_H_ (*p*-Value)	Egger (*p*-Value)
0.366	0.051	0.267–0.466	0.0%	0.812	-

## Data Availability

Not applicable.
